# The Applications of 3D Printing for Craniofacial Tissue Engineering

**DOI:** 10.3390/mi10070480

**Published:** 2019-07-17

**Authors:** Owen Tao, Jacqueline Kort-Mascort, Yi Lin, Hieu M. Pham, André M. Charbonneau, Osama A. ElKashty, Joseph M. Kinsella, Simon D. Tran

**Affiliations:** 1 McGill Craniofacial Tissue Engineering and Stem Cells Laboratory, Faculty of Dentistry, McGill University, 3640 University Street, Montreal, QC H3A 0C7, Canada; 2 Department of Bioengineering, McGill University, 817 Sherbrook Street West, Montreal, QC H3A 0C3, Canada; 3 Department of Orthodontics, Guanghua School of Stomatology, Hospital of Stomatology, Sun Yat-sen University, 56 Lingyuan Road West, Guangzhou 510055, China; 4 Oral Pathology Department, Faculty of Dentistry, Mansoura University, Mansoura 22123, Egypt

**Keywords:** 3D printing, additive manufacturing, bioprinting, dentistry, oral and maxillofacial regions, tissue engineering

## Abstract

Three-dimensional (3D) printing is an emerging technology in the field of dentistry. It uses a layer-by-layer manufacturing technique to create scaffolds that can be used for dental tissue engineering applications. While several 3D printing methodologies exist, such as selective laser sintering or fused deposition modeling, this paper will review the applications of 3D printing for craniofacial tissue engineering; in particular for the periodontal complex, dental pulp, alveolar bone, and cartilage. For the periodontal complex, a 3D printed scaffold was attempted to treat a periodontal defect; for dental pulp, hydrogels were created that can support an odontoblastic cell line; for bone and cartilage, a polycaprolactone scaffold with microspheres induced the formation of multiphase fibrocartilaginous tissues. While the current research highlights the development and potential of 3D printing, more research is required to fully understand this technology and for its incorporation into the dental field.

## 1. Introduction

Three-dimensional (3D) printing is an emerging additive manufacturing technique capable of building complex 3D geometric structures, which can be used as scaffolds for craniofacial tissue engineering including the fabrication of biocompatible polymeric implants, the replication of intricate matrix geometries, and the development of biodegradable scaffolds, to cultivate transplantable tissues or organ replacements.

3D printing technology started in 1990, and it was mainly focused on fabricating scaffolds constituted of synthetic inks. It was not until the last decade when the technique evolved to what we currently know as bioprinting [1,2]. The development of bioinks, biocompatible soft materials that contain biological components such as cells or naturally derived matrices, promoted tissue engineering applications [3,4].

Several studies have successfully developed structures with relevant characteristics for regenerative dentistry—hydroxyapatite (HA) modified hydrogels have been reported suitable for bone bioprinting due to their osteosupportive and osteoinductive properties [5]. Additionally, polymers such as polycaprolactone (PCL) have also been reinforced with HA particles and 3D printed, resulting in scaffolds with good bioactivity shown by their in vitro apatite-forming ability [6].

Current bioprinting techniques include inkjet, stereolithography, laser-induced forward transfer (LIFT), and extrusion [7]. All of them can encapsulate cells or particles of interest in the hydrogel bioink. After printing, all these techniques provide cells with a 3D environment that mimics the biological conditions found in vivo. These techniques allow the design and fast fabrication of multiple models with the same architecture and dimensions of the original design with variables that can be easily controlled and manipulated for experimental purposes.

3D printing is a novel technique that is rapidly evolving and can become an important tool for the development of tissue-like constructs for oral surgery or translational research of biology and disease in dentistry. In this review, we will examine the types of 3D printing methodologies and materials as well as the applications of 3D printing in specific fields of dentistry such as the periodontal tissues, dental pulp, bone, and cartilage.

## 2. Three-Dimensional Printing Methodologies

### 2.1. Inkjet Printing

**Figure 1:**
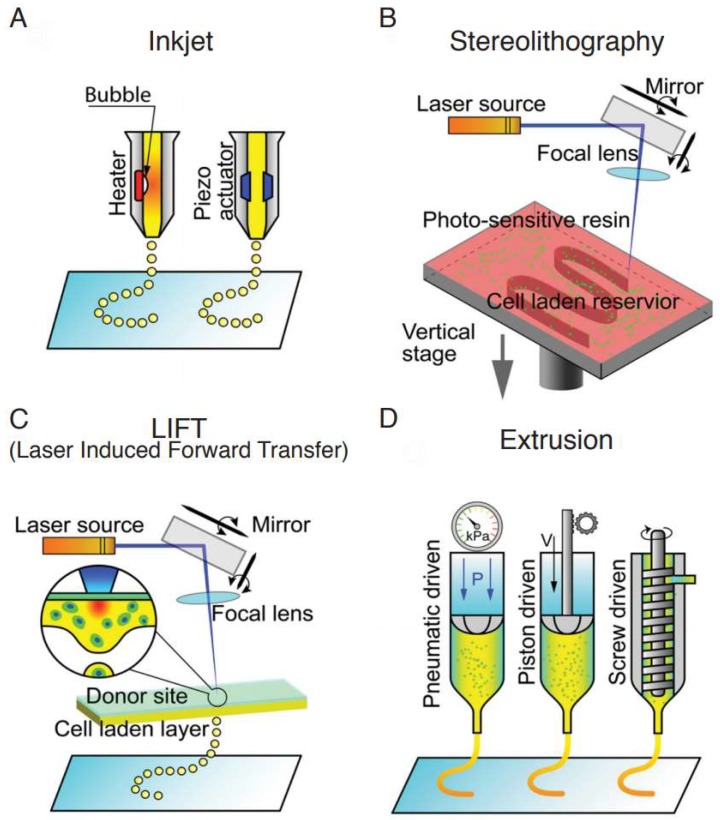
Schematic of various 3D printing methodologies. (**A**) Inkjet. A heater or piezo actuator deposits droplets. (**B**) Stereolithography. Layer by layer photopolymerization of a liquid resin by laser. (**C**) Laser induced forward transfer. Droplets of the material induced by a laser source. (**D**) Extrusion. Material exiting a nozzle that is pneumatic, piston, or screw driven. Reproduced with permission from [7].

The principle behind inkjet printing consists of introducing a small volume change upstream of the nozzle, creating a pressure change, which results in a droplet ejection downstream (Figure 1A). This is performed by an inkjet head system which can be either piezoelectric or thermal induced. Thermal induced heads use a resistor as their heating structure. When current passes through the resistor, the fluid in contact is vaporized, creating a bubble that expands in the reservoir. This increases the pressure causing a droplet ejection through the nozzle [8]. Piezoelectric heads cause the volumetric change by applying a voltage pulse to the piezoelectric material [9].

A wide range of powder materials such as polymers, ceramics, proteins, and cells can be processed using this technique. However, the ink’s viscosity is limited to 5–20 Pa.s to avoid high ejection pressures or continuous flow of the material. The main advantages of this technique include high-speed printability, low cost, and the possibility to encapsulate cells in the material [10].

For bone regeneration, bioink patterns using inkjet printing have been studied to control osteoblast differentiation in vitro and bone formation in vivo. A study created patterns of bone morphogenetic protein-2 (BMP-2) within microporous scaffolds (made from DermaMatrix, containing various extracellular molecules such as collagen and fibronectin) and observed cell differentiation in vitro and tissue formation in vivo in the patterned areas [11]. Additionally, another study used inkjet printing to 3D print calcium phosphate scaffolds and incorporated a collagen coating. The implanted scaffolds were osteoconductive while being biodegradable [12].

### 2.2. Laser-Assisted 3D Printing 

Although less commonly used, laser printing technology has emerged from LIFT technology as a promising method for tissue engineering. It has prominent advantages in terms of bioprinting and is also known as laser-assisted bioprinting (LAB). LIFT assisted printers or LAB basically have three main components: (1) a pulsed laser source, (2) a target serving as a support for the printing materials, usually a transparent glass slide or ribbon, and (3) a receiving substrate to collect the materials. During printing, a focused laser pulse stimulates a small area of the target, which comprises an energy-absorbing layer on the surface and bioink solution underneath. Then a portion of the energy-absorbing layer is evaporated, resulting in the formation of a droplet that is collected by the receiving substrate and crosslinked therein [13,14].

Unlike inkjet printers, laser-assisted printers are equipped with no nozzles, obviating direct contact between the dispenser and the bioinks and therefore minimizes the problem of materials/cells clogging. With that, they are compatible with more materials, especially those with high viscosity (1–300 mPa/s), and can maintain cell viability higher than 95% [15]. Those benefits along with products with higher resolution makes laser-assisted printing a promising technology for tissue engineering.

Variations of laser-assisted 3D printing include selective laser sintering (SLS), stereolithography (SLA) (Figure 1B) and LIFT (Figure 1C). In particular, SLS has been extensively used for the regeneration of tissue with complex anatomy like craniofacial bone or cartilage. Developed by Carl Deckard for his Master’s thesis at the University of Texas in 1989, SLS uses a high-powered carbon dioxide laser to create structures by fusing the powder layer-by-layer with the underlying powder as support [16]. The laser beam fuses powders selectively on the basis of sectional data from computer-assisted design (CAD). After a layer is created, the powder bed descends and another layer is rolled over. Such process is repeated until the scaffold is completed [17]. SLS can produce tissue engineering scaffolds from a variety of powder materials, including metals, bio-ceramics, and synthetic polymers like polylactic acid (PLA), PCL, poly ethyl ether ketone (PEEK), and poly ether ketone ketone (PEKK) [18,19]. Some researchers also include HA powders in the polymers to increase the osteoinductivity of bone scaffolds [20]. Natural polymers cannot be utilized with this technique due to the high temperatures generated by the laser during printing. However, similar to growth factors, they can be incorporated into the scaffolds post-printing.

PCL is an advantageous material in SLS, mainly because of its low melting (59–64 °C) and glass-transition temperatures (−60 °C) that facilitate the prototyping process. SLS-printed PCL scaffolds have been used to repair the periodontal [21], craniofacial bone, or osteochondral defects [22,23,24], and were proved to be biocompatible with adequate strength. Another polymer that has been recently applied in craniofacial regeneration using the SLS technique is PEEK [19], which has more favorable mechanical properties for stress loading than PCL. The manufacturing strategy, however, is not much different from that of PCL scaffolds. Higher performance polymers like PEKK have also been successfully printed by SLS technique [25,26] and introduced in the application of the craniofacial area [27,28].

Nowadays, no polymer processing methods can compete with the SLS regarding the fabrication flexibility and complexity of the 3D shapes obtained. However, the advancement of laser-assisted technologies is obviously restricted by the complicated control of the laser printing system and concerns about the side effect of laser exposure.

### 2.3. Extrusion 

Fused deposition modeling (FDM) is the most common 3D printing technique. It uses a continuous filament made of thermoplastic polymer that is melted at the nozzle into a semi-liquid state and then extruded on a platform or on top of the previous layer (Figure 1D). The material fuses together to create a continuous structure after solidifying at room temperature. The quality of the extruded filament may be modified by adjusting the printing velocity, layer thickness, and printing orientation [29]. Some of the most common materials used for this technique are polycarbonate (PC), acrylonitrile butadiene styrene (ABS), PCL, and PLA due to their low melting points compared to other thermoplastics [30].

The main advantages of this technique are the affordability, high-speed printing, and the potential of printing multiple materials at the same time when working with a multi-nozzle printer [29]. Some disadvantages include the limitation to use only thermoplastic materials and the inability to embed cells in the material since thermoplastics melt at temperatures higher than 37 °C.

FDM-printed coated scaffolds and composite materials have been proven as useful tools for bone tissue engineering and regeneration. PCL scaffolds with freeze-dried platelet-rich plasma (PRP) were implanted in rats, and the results show that it can promote osteogenic differentiation of dental pulp stem cells and induce bone formation [31]. Additionally, anatomically shaped molar scaffolds made of PCL and hydroxyapatite with 200-µm-diameter interconnecting microchannels were implanted in rats and growth factors (stromal-derived factor-1 (SDF1) and bone morphogenetic protein-7 (BMP7)) were perfused. This setup recruited more endogenous cells and generated more angiogenesis than the control group [32].

Three-dimensional plotting (3DP) is a technique very similar to FDM. It consists of extruding a viscous material from a cartridge using pneumatic or mechanical pressure through a nozzle onto a defined position in a platform [30]. Multiple cartridges are mounted in an XYZ stage and the position of each cartridge, the pressure, and temperature are controlled by a computer. As with FDM, this technique also allows the printing of heterogeneous structures with different materials. Printing and curing of the materials is also possible by extruding the reactive components using mixing nozzles, exposing each layer to UV light or heating the stage to stabilize the material after printing [33]. The material flexibility is the main advantage of this technique. Hydrogels, plastics, pastes, and solutions can be printed using this technique, and several of these can be biocompatible allowing cell encapsulation before printing. Some disadvantages of this method compared with FDM are the resolution and speed.

Multiple bioinks suitable for this technique have been proposed to promote bone tissue regeneration. Studies using 3D printed periodontal cells encapsulated in a bioink constituted of different ratios of gelatin methacrylate (GelMA) and poly (ethylene glycol) (PEG) dimethacrylate have proven useful to study periodontal ligament stem cell response to extracellular matrix components [34]. Additionally, polymer solutions based on methacrylated gelatin and methacrylated hyaluronic acid modified with HA particles were used to encapsulate human adipose-derived stem cells and bioprint structures, which proved to be a suitable material for bone bioprinting applications [5]. A summary of the 3D printing types and their potential applications can be found in Table 1.

## 3. Materials for Three-Dimensional Printing

### 3.1. Polymers

Polymer materials are composed of chemical compounds typically formed from carbon, hydrogen, oxygen, and nitrogen. These monomer structures are repeated and bound with itself to create a longer molecular chain [35]. Polymers have become the most popular choice for 3D bioprinting in biomedical applications as it is often inexpensive, biocompatible, biodegradable, and can easily be manipulated with regard to its mechanical, chemical, and biological properties [36,37]. The polymer’s manipulative property is particularly important in 3D printing because the printability of a material is dependent on its viscosity [37]. The ink being printed should be stiff enough to support subsequent layers, however if it becomes too viscous, it may lead to blockage of the printing nozzle [37]. Blockage is further avoided by printing the material in its pre-polymerized form. However, a limitation to using synthetic polymers is that the printing process for synthetic polymers induces high temperatures in which cells and growth factors cannot survive or remain active. Thus, cells or biologically active components are incorporated post-extrusion [37]. 

Polymeric materials can be printed in various forms as well, including powder, filament, and sheet form [38]. The polymer category encompasses a wide variety of materials that can range from being soft to hard, or synthetic to natural, however, the most commonly used polymers in craniofacial tissue engineering include PCL, PEEK, PLA, poly(lactic-co-glycolic acid) (PLGA), and chitosan [2,39,40]; the choice of polymer will depend on the goal of the researcher. For example, PLA or PCL are popular choices for drug delivery purposes, while alginates and gelatin are more popular choices for cell encapsulation [37,38]. The wide variety in polymeric materials makes them highly versatile, and thus may be highly useful in dental tissue regeneration when being combined with the superior spatial resolution provided by 3D printing.

### 3.2. Ceramics 

Ceramic materials consist of metals with inorganic calcium or phosphate salts (such as calcium silicate or β-tricalcium phosphate) and are generally osteoconductive and osteoinductive [41]. The composition of these scaffolds also allows them to last longer than hydrogels, permitting more time for structural support and for guided tissue regeneration. Although the properties of ceramic materials allow for cells to quickly proliferate and differentiate on the scaffold, a limitation lies in its inherent brittleness and poor mechanical strength—characteristics that may be necessary when dealing with load-bearing defects [41]. Several studies have however aimed to improve the effectiveness of ceramic materials by changing the pore size as well as through the addition of polymers, such as PCL or PLA. These alterations to the ceramic-based materials allow them to resemble better the mechanical properties of natural bone while being able to promote vascularization [42].

In a review by Jammalamadaka and Tappa, various 3D printing methodologies (such as extrusion [43,44,45], inkjet [12,46,47], and laser sintering [48,49]) for ceramic-based materials are mentioned, in addition to the use of sintering and freeze-drying methods post-printing to improve the mechanical properties of the scaffolds [42]. The FDM printing of ceramics is briefly outlined in a review by Obregon and colleagues, where scaffold manufacturing consists of three phases that use organic particles to facilitate flowability, which are then burned out with high temperatures leaving behind primarily the inorganic ceramic particles [37].

### 3.3. Composites 

Printable composite materials are composed of a minimum of two different materials; mixtures for printable composites being used in dentistry are typically composed of copolymers, polymer-polymer mixtures, or polymer-ceramic mixtures; be ceramic-based or hydrogel-based; and can include the addition of biomolecules, carbon nanotubes, and metals [37,50,51]. The mixture will be dependent on the goal of the composite, but it is typically created to manipulate ink properties such as processability, printability, stiffness, and bioactivity [51]. By combining multiple materials, composite materials can harness the benefits of each individual material [50]. For example, the polymer PLA alone has great chemical and physical properties, however, may not be optimally biocompatible as it releases acidic compounds over time. Researchers overcame this issue by creating a composite containing PLA and ceramics such as calcium phosphate, which ultimately lessens the formation of acidic environments formed by PLA [51]. Composites can also be enhanced with silicate fillers and nanoparticles, which alter its viscosity and stiffness and ability to influence cell morphology [37]. 

Composite materials are frequently used in craniofacial regeneration due to its unique properties. While hard polymers exist, composite materials are more capable of mirroring complex tissues that withstand higher mechanical stress and loads such as bones and teeth, and thus are more favorable for craniofacial regeneration [37]. For example, in bone regeneration, researchers again have combined PLA with ceramic to take advantage of PLA’s mechanical properties while overcoming its brittle nature [51]. Other uses for printable composites include cartilage regeneration and whole-tooth regeneration [37].

### 3.4. Cell Aggregates 

Bioprinting of cell aggregates has been used as a scaffold-free methodology of creating tissue engineered constructs. These cell aggregates consist of spheroid structures, which can then be specifically positioned, creating for instance tubular or ring-like structures [52,53]. Although the constructs are primarily scaffold-free, the cells are usually encapsulated with a hydrogel material that is biocompatible and biodegradable, for cell survival and for mechanical support of the cell construct. The hydrogel also helps to prevent tissue fusion while the cells are maintained in the suspension reservoir of the 3D printer [54]. The use of cross-linking solutions, such as those containing CaCl_2_ or gelatin, can help to further minimize cell aggregation [55,56]. As the pH of the bioink is important for cellular survival and scaffold integrity, a study by Lozano and colleagues used the addition of NaOH to stabilize the pH of a modified bio-polymer hydrogel [57]. 

The advantages of using scaffold-free constructs for tissue engineering include the absence of potentially toxic or immunogenic scaffold materials, as well as the ability to create high cell density constructs [37,58]. Limitations of the cell aggregate approach include the relatively time-consuming cellular fusion of the spheroids to create larger tissue structures (which may also create non-uniform structures) [54]. Certain advances have been made to minimize this limitation such as the development of multicellular cylinders as an alternative structure, which require up to four days to create the appropriate shape [53]. While most 3D printing of cell aggregate studies have been performed in vitro, there is a limited understanding of its potential in vivo and further studies must be performed to demonstrate its safety and feasibility as a scaffold-free construct for tissue engineering. A summary of the 3D printing materials and their potential applications can be found in Table 2.

## 4. Pre-Clinical and Clinical Applications

### 4.1. Periodontal Complex 

The concept of using 3D printing for periodontal tissue regeneration is to guide locally available cells to restore periodontal defects, termed guided tissue regeneration (GTR) [59]. These cells can use the support of a 3D printed scaffold, as well as surrounding growth factors, bioactive proteins, etc., to regenerate damaged periodontal tissues [60]. While epithelial tissues regenerate quickly, bone tissues require more time, and therefore a difficulty in periodontal regeneration lies in its tissue complexity (cementum, periodontal ligament, etc.) [59]. To address these challenges, the creation of multiphasic scaffolds has allowed for various properties within a scaffold, which mimics better the composition of the native periodontal complex [59].

PCL scaffolds have been 3D printed and used for periodontal tissue engineering. Improvements to these scaffolds have focused on several key aspects, as stated by Ivanovski and colleagues, relating to periodontal tissue engineering: (1) compartmentalized bone and periodontal attachment tissue formation; (2) cementum formation onto the root surface; (3) correctly oriented periodontal ligament fibers [61]. Lee and colleagues, for instance, 3D printed PCL-HA scaffolds with varying sizes of microchannels to create a compartmentalized multiphasic scaffold [62]. FDM was used to create these scaffolds, which had 100 μm microchannels designed for the cementum/dentin interface, 600 μm for the periodontal ligament (PDL), and 300 μm for the alveolar bone [62]. They found that in vivo implantation of dental pulp stem cells with the scaffold resulted in differentiation of the cell population into putative dentin/cementum, PDL, and alveolar bone [62]. Similarly, Li and colleagues used a freeze-dried PRP coating to improve the biological properties of PCL scaffolds [31]. This coating was applied to the 3D printed PCL scaffold for 5 min at room temperature, then placed at −80 °C for 30 min, followed by freeze-drying [31]. The freeze-dried PRP-PCL scaffolds induced significantly greater bone formation compared to traditional PRP-PCL or bare PCL scaffolds [31]. 

Bioprinting of PDL cells, creating a 3D hydrogel microarray, has been performed to screen for cell-biomaterial interactions [34]. The cells were bioprinted using a pressure-assisted valve-based bioprinting system placed within a sterile hood and controlled by a computer [34]. The pressure-based system replaces the need for any external stimulation, and thereby minimizes shear forces and high temperatures, allowing for the cells to survive the printing process [34]. This study has found that the viability of the printed periodontal cells was maintained throughout printing, and therefore this methodology can perhaps be used to 3D print periodontal cells directly into future scaffolds [34]. Likewise, Hamlet and colleagues examined alveolar bone regeneration through 3D hyaluronic acid hydrogels containing osteoblasts and found that their hydrogel provided a favorable environment and could stimulate osteogenic gene expression in vitro: they believed that this hydrogel could be optimized as a cell-delivering bioink for future 3D bioprinting applications [63].

**Figure 2:**
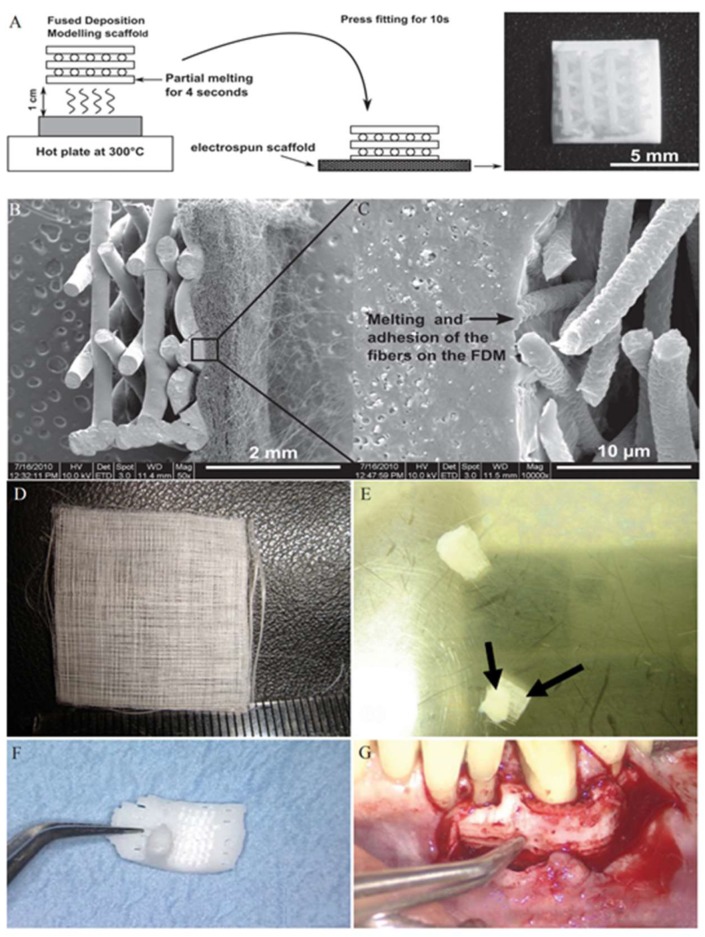
3D Printed scaffolds for periodontal tissue engineering. (**A**–**C**) Schematic of the scaffold fabrication methodology (**A**). Cross-section showing the fusion of the electrospun fibers with the fused deposition modeling (FDM)-printed compartment of the scaffold (**B**,**C**). (**D**,**E**) Electron spun polycaprolactone (PCL) scaffold (**D**). The PCL scaffold attached to a decellularized sheet (**E**). (**F**,**G**) selective laser sintering (SLS)-printed PCL scaffold to be implanted in patient (**F**). Scaffold placement for implantation (**G**). Reproduced with permission from [21,65,67].

The combination of cell sheets with 3D printed scaffolds has also been used for periodontal tissue regeneration. Vaquette and colleagues cultured PDL cell sheets in 24-well plates, which were then combined with an FDM printed PCL scaffold by folding the cell sheet over the scaffold (Figure 2A–C) [64]. They found that the scaffolds incorporating the cell sheet technology had better attachment onto a dentin surface than those without [64]. Farag and colleagues, however, aimed to improve the cell sheet technology by decellularizing the cell sheet after combination with the PCL scaffold (Figure 2D,E) [65]. The decellularization aimed to use the properties of the PDL extracellular matrix to promote periodontal regeneration, while minimizing the immunogenic effects of cellular material. They found that the decellularized cell sheet constructs upregulated the expression of mineralized tissue markers in PDL cells [65]. As an application of scaffold free bioprinting, Bakirci and colleagues developed a novel cell sheet based bioink for 3D bioprinting [66]. Cells were first grown on poly(N-isopropylacrylamide) coated surfaces, harvested, and centrifuged into cell sheet aggregates to be used for bioprinting applications [66]. Although this study developed a cell sheet based bioink using human skin fibroblasts, it illustrates the possibility in developing a PDL cell sheet based bioink, which could then be used for periodontal tissue engineering applications such as scaffolds in treating periodontal-related cases. 

Clinically, Rasperini and colleagues reported the use of a SLS printed PCL scaffold to treat a periodontal defect (Figure 2F,G) [21]. A computed tomography scan of the patient’s defect was taken to modify the scaffold design and to create a customized scaffold [21]. This scaffold consisted of an internal port for growth factor delivery and pegs perpendicular to the root to facilitate PDL formation [21]. At two weeks, the scaffold was removed and unfortunately the patient showed minimal evidence of bone repair [21]. However, this case highlighted a potential for the use of 3D printed scaffolds in treating periodontal-related cases. 

### 4.2. Dental Pulp

The dental pulp is an unmineralized tissue beneath the mineralized hard exterior of the tooth, which plays a crucial role in tooth vitality; injury, alteration, and/or removal of the pulp may lead to tooth necrosis. Additionally, the pulp may also service to provide immunity, nutrition, and sensation as well [50]. As a result, there is a need to focus on protecting the pulp from trauma, and to regenerate the pulp should it be injured. While there has not been major success in pulp regeneration due to the challenges met in nurturing, revascularizing, and reinnervating the pulp tissue, a promising direction that researchers are exploring is the use of hydrogels to contain and nurture dental pulp cells [68]. By using hydrogels and other biomaterials as cellular scaffolds to mirror the native in vivo environment, researchers can promote cell growth, differentiation, and morphogenesis [50]. However, the major constraint with using hydrogels alone is that spatial manipulation is limited, i.e., researchers cannot fully control multicellular organization and interaction, thus the overall morphogenesis of the artificial gland or tissue. By applying the superior spatial control of 3D cell printing, this issue can be overcome in pulp tissue regeneration [69]. 3D cell printing technology would enable researchers to suspend and position various cells contained in hydrogels as they desire. For example, researchers could print odontoblastoid cells along the dentin walls while having fibroblasts towards the center of the pulp chamber [69]. Furthermore, the enhanced precision gained from 3D bioprinting would allow researchers to achieve specific cellular interactions, anisotropic mechanical properties, and desired distribution of growth factors [70]. While theoretically the use of 3D cell printing in pulp tissue regeneration sounds feasible, there is a lack of evidence for this to date. Several studies have shown the possibility of successfully 3D printing blood capillaries, however in vivo angiogenesis has not been exhibited in endodontics [37,69].

While there is a lack of in vivo studies to date, there are several studies that highlight the potential use of 3D cell printing in dental pulp regeneration. For example, in a study by Athirasala and colleagues, they showed that the mouse odontoblast-like cell line (OD21) could be supported in a novel hydrogel composed of alginate and dentin (Alg-Dent) [71]. While this study did not address more complicated experiments such as the possibility of vasculogenesis and/or angiogenesis, the use of human cells, and cell survivability in root canals and chamber, the study demonstrated the tunability and printability of the scaffold using 3D bioprinting technology. The success of this paper indicated the potential of the scaffold and how 3D bioprinting could further enhance the feasibility of this hydrogel in regenerative endodontics. Specifically, researchers would be able to localize growth factors and other nutrients precisely to the desired targets such as the peripheral dentin or central pulp to induce cell-specific regeneration as evident in this study.

**Figure 3:**
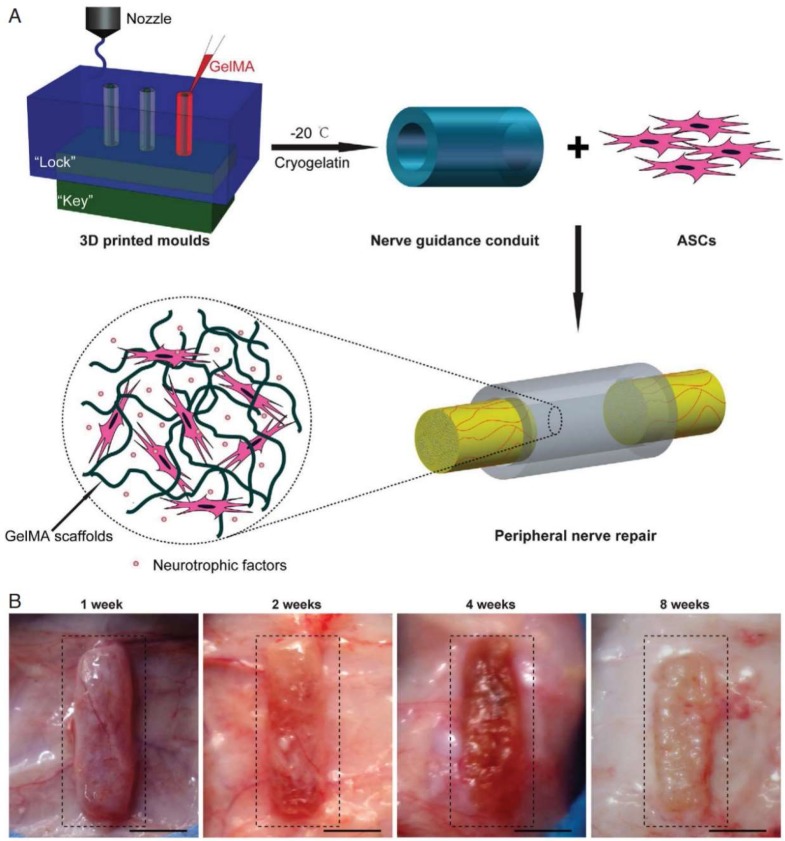
Cellularized conduits for peripheral nerve regeneration created using 3D printed molds. (**A**) Schematic of the conduit fabrication method. (**B**) Photographs of the rat dorsal side with the biodegradable nerve guidance conduit positioned subcutaneously. The figures are © 2016, Hu Y., Wu Y., et al. (https://doi.org/10.1038/srep32184) used under a Creative Commons Attribution 4.0 International License: http://creativecommons.org/licenses/by/4.0/.

As previously mentioned, 3D bioprinting methods allow researchers to achieve superior tunability, creating scaffolds that would not be possible without it. For example, in a recent study by Feng and colleagues, they compared two different techniques in fabricating a PLA scaffold, either molding via standard extrusion processes or 3D printed, and its influence on dental pulp cells. The results indicated that manufacturing techniques can influence differences in cell migration, morphology, and differentiation marker expression [72]. Another study by Hu and colleagues used 3D printed molds to create cellularized conduits for peripheral nerve regeneration, which showed comparable results to the use of autografts in repairing peripheral nerve defects (Figure 3) [73]. Thus, further studies should be explored, comparing current scaffold manufacturing methods and 3D printing and its effects on vasculogenesis and angiogenesis in support of pulp regeneration.

Though there are not many studies to date highlighting the use of 3D printing in pulp regeneration, the current studies that demonstrate the superior tunability and modifications in the mechanical properties of currently viable scaffolds using 3D printing indicates the potential it may have in pulp regeneration. Further studies need to be explored by implementing the results, knowledge, and support gained from current studies in the possibility of inducing vasculogenesis, angiogenesis, and nutritional support in pulp tissue using 3D printing techniques.

### 4.3. Cranio-Maxillofacial Tissues

Craniofacial bones and cartilages comprise the craniofacial skeleton that impart specific appearance and function. It is challenging to reconstruct craniofacial structures due to the complex 3D geometry. With the combination of image-based extraction of craniofacial geometry and the ability to 3D-print shapes with high fidelity, 3D printing technologies are ideally suited for the manufacture of bone and cartilage scaffolds tailored to specific defects. The goal of craniofacial 3D reconstruction is to mimic the external and internal architecture of the host site and to provide essential framework for cell attachment and migration.

Bone is considered the second most transplanted tissue for defects due to trauma, osteoporosis, bone tumors, etc. Many types of biomaterials have been proposed to integrate the desirable properties, such as biocompatibility, printability, osteoconductivity, osteoinductivity, and mechanical properties, attempting to mimic the natural replacement of bone.

Bioceramics are the most commonly selected materials. They are usually composed of calcium and phosphate mineral phases, such as HA, β-tricalcium phosphate (TCP), or bioactive glasses (BGs). They exhibit outstanding biocompatibility and favorable biodegradability. Moreover, 3D printed ceramics can upregulate osteogenesis by creating a bioactive ion-rich cellular micro-environment and promote cell proliferation by close cell-cell interactions [74]. Even so, ceramic scaffolds are too brittle for implantation in load bearing craniofacial sites. Saijo and colleagues have confirmed this disadvantage by using HA/α -TCP composite scaffolds for maxillomandibular defects, which showed difficulties in composition and fabrication of an ideal scaffold to fulfill strength and dimensional requirements [75]. Still, Shao and colleagues have recently reported that ~10% Mg-substituted wollastonite had much higher flexural strength (31 MPa) than TCP and other calcium-silicate porous bioceramics [76]. By adding a range of metallic ions like Cu^2+^ and Co^2+^ into BGs, the angiogenic activity in vivo can be developed, which is beneficial for the healing process [77]. Compared to being used alone, they are more commonly incorporated with other biomaterials such as polymers for the enhancement of osteogenesis and osteoinductivity.

**Figure 4:**
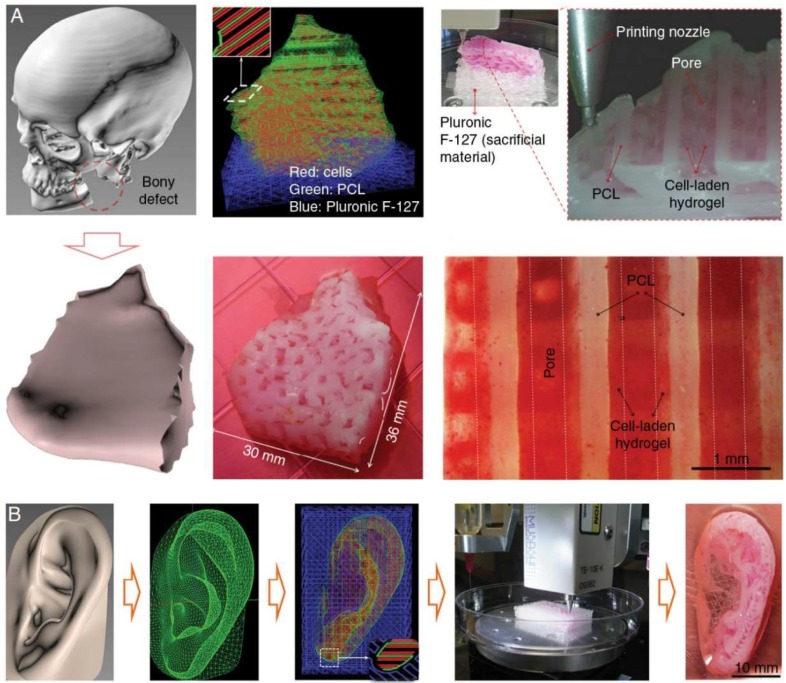
Craniofacial bone and cartilage reconstruction using PCL as a material for 3D printing. (**A**) Mandibular bone reconstruction. 3D defect model was obtained from the craniofacial CT image data followed by the design of dispensing paths of cells, PCL, and Pluronic F-127 with self-developed software. Multiple cartridges used to deliver and pattern the above ink materials were connected to a microscale nozzle, which dispensed the materials according to the design during 3D printing process. PCL was printed as the framework and the cell-laden hydrogel were dispensed to fill the pores, while Pluronic F-127 were used as sacrificing materials. The osteogenic potential of the scaffold was confirmed by Alizarin Red S staining after being cultured in osteogenic medium for 28 d. (**B**) Auricle cartilage construction. Similarly, a 3D computer-assisted (CAD) model of auricle can be developed from CT or MRI image data and generate a visualized motion program consisting of a command list for XYZ stage movements and air pressure actuation for 3D printing. The concentrations of different ingredients for 3D printing can be optimized by in vitro culture and related tests. Reproduced with permission from [90].

Polymers are another widely-used material that is superior in its printability and efficiency in promoting osteogenesis. The main concerns are its poor cellular interaction and low stiffness. PLA and PGA, for example, are now rarely used for bone scaffolds considering their low compressive strength and osteoconductivity. However, their co-polymer PLGA and another polyester, PCL, have remarkable osteoconductivity and better mechanical properties. By comparison, PCL has a lower rate of degradation and subsequently denser tissues generated [78]. Therefore, it is preferred to be used as the framework of composite scaffolds. As PCL is bioinert, other biological active components such as TCP, HA, decellularized trabecular bone, or growth factors were incorporated into the 3D printing system [21,79,80]. Furthermore, the acidic environment caused by the degradation products of PCL and its hydrophobic nature can be somewhat diminished by the inclusion of hydrophilic polymers like PEG and the surface coating of natural polymers like chitosan [81,82]. PCL scaffolds are well-suited for extrusion-based 3D-printing (Figure 4A), FDM for example, due to the relatively low melting points (62 °C). In recent decades, SLS has been developed as a more precise 3D printing technique that can fine-tune the porosity to optimize conditions for cell growth and proliferation. Additionally, a wide range of thermoplastic materials including high performance plastics with specific mechanical properties can be processed by SLS technique. The stiffness of PCL scaffolds manufactured by SLS has been reported to be ~15 to 300 MPa, values that are much higher than conventional 3D polymers but still lower than human trabecular bone within the condyle (120–450 MPa) or within the mandibular body (112–910 MPa) [22]. Metal-based scaffolds, on the other hand, are stiff enough but possess a considerably higher Young’s modulus, which would lead to stress shielding issues and therefore failure of the implants. Polyaryletherketones (PEAKs) is a family of high-performance polymers with compatible Young’s modulus to natural bone, which would be a suitable property for load bearing orthopedic and craniofacial implants. PEKK is by far a material with the most advantageous performance in the PEAKs family. The PEKK printed by SLS platforms have showed desirable mechanical properties, great biocompatibility, and osteointegration in a craniofacial bone defect model in vivo [18,27,28]. As a promising 3D printing material for bone tissue engineering, more evidence of success is needed for future applications.

The cartilaginous tissues in the craniofacial area primarily include the temporomandibular joint (TMJ) disc, the auricle cartilage, and the nasal cartilage. The bioinks used for cartilage reconstruction should be able to mimic the 3D architecture with mechanical anisotropic, nonlinear, and viscoelastic behavior analogous to native cartilage.

In early approaches, many hydrogels encapsulating the chondrocytes/mesenchymal stem cells (MSCs) with the capability of synthesizing extracellular matrix were fabricated by micro-extrusion technique. The cell-laden hydrogels ranged from natural polymers like alginate and collagen to synthetic polymers like gelatin metacrylamide (GelMA) and polyethylene glycol dimethacrylate (PEGDMA) [83,84,85,86]. To improve the mechanical properties, higher polymer concentration and viscosity were preferred. On the other hand, cells proliferate and differentiate towards cartilage tissue more readily within lower polymer concentrations. This dilemma increased the challenge of using the hydrogels alone to reconstruct the cartilaginous tissue. The most common solution is to include a stiffer thermoplastic polymer such as PCL to cell-laden hydrogels by coextrusion or other hybrid strategies (Figure 4B). PCL acts as a frame to reinforce the constructs, and by modulating the polymer percentage, the compressive equilibrium moduli in the range of articular cartilage can be achieved [87,88,89].

The degradation rate of PCL, which can be up to 2–3 years, is a potential limitation with such multi-material approaches, as residual filaments can act as a barrier to tissue formation. One alternative is applying polymers that have a higher rate of degradation, poly (hydroxymethylglycolide-co-caprolactone) (PHMGCL) or PLGA, for instance [91]. Yet the acidic by-products of PLGA that cause adverse inflammatory response still remains a concern for future applications. Tarafder and colleagues developed a region-variant TMJ disc scaffold by incorporating the specifically-aligned PCL with PLGA microspheres encapsulating TGFβ3 [92,93]. After seeding with MSCs, multiphase fibrocartilaginous tissues formed and significantly improved the healing process of the perforated disc. The dynamic function was also restored as no arthritis changes were observed on the condyle four weeks post-implantation.

Another way to reduce the residual PCL materials is to increase its porosity by the melt-electrowriting (MEW) technique, which is similar to FDM but using a nozzle tip equipped with voltage. PCL fabricated by MEW can be very thin, with a diameter down to 0.8 µm, and therefore the porosity can be high, up to 93%–98% [94]. In addition, the stiffness and yielding strains of the resultant scaffolds were within the range of native cartilage.

## 5. Conclusions

3D printing has the potential to revolutionize dentistry. This technique allows for a layer-by-layer construction of tissue engineering scaffolds—to create accurate, yet complex scaffold models for personalized patient treatments. Recently, there have been many advances in 3D printing for dentistry: for the periodontal complex, FDM printed scaffolds have been modified to induce greater bone formation, while an SLS printed scaffold has been applied for the first time in a human patient; for dental pulp, a 3D printed hydrogel could support odontoblast cell survivability; for bone and cartilage, modified bioceramic scaffolds induced greater angiogenesis and a modified PCL scaffold induced greater fibrocartilaginous tissue formation. While there are many in vitro studies examining the efficacy of 3D printed scaffolds for tissue engineering, further research must be performed to better understand the potential of these scaffolds in vivo, and to address any unprecedented safety concerns. As this technology develops, we expect to see a greater number of dental offices equipped with 3D printing technology, not only for the printing of crowns and dentures, but also for the purposes relating to tissue engineering.

## Figures and Tables

**Table 1:** Summary of 3D printing types.

Type	Methodology	Applications
**Inkjet**	Pressure change upstream of nozzle resulting in a downstream droplet ejection.	Regenerative approach—Printing of complex ceramic-like structures to support guided tissue regeneration. Replacement approach—Drop-by-drop bioprinting of live cells for the cell aggregate approach.
**Laser-Assisted**	Laser pulse stimulates a small area of the target.	Regenerative approach—Creation of more complex scaffolds for guided tissue regeneration.
**Extrusion**	Material fuses together at room temperature after leaving the nozzle.	Regenerative approach—Can be used with many materials for the creation of simple biocompatible and biodegradable scaffolds for guided tissue regeneration.

**Table 2:** Summary of 3D printing materials for tissue engineering.

Type	Materials	Applications
**Polymers**	Compounds typically formed from carbon, hydrogen, oxygen, and nitrogen, such as PCL, PEEK, PLA, PLGA.	Regenerative approach—Uses biodegradable polymers as a guide for tissue regeneration.
**Ceramics**	Metals with inorganic calcium or phosphate salts (calcium silicate or β-tricalcium phosphate).	Regenerative approach—Longer-lasting ceramic-type scaffolds can permit more time for structural support and for guided tissue regeneration.
**Composites**	A combination of a minimum of two different materials, for instance copolymers, polymer-polymer mixtures, or polymer-ceramic mixtures.	Regenerative approach—Composites (such as PLA with ceramics) can be created to facilitate the regenerative approach by reducing the formation of acidic environments caused by PLA alone. Replacement approach—Composite hydrogels (such as those containing silica) can be created to facilitate the replacement approach by increasing gene expression of BMPs.
**Cell Aggregates**	Cell aggregates form spheroid structures, which are then used as a scaffold-free application of tissue regeneration.	Replacement approach—Post-printing fusion of spheroids create structures that can be used as replacements for damaged or missing tissues.
